# A historical review of promotions of physical activity for adolescents in China from 1949 to 2020

**DOI:** 10.3389/fpubh.2024.1415513

**Published:** 2024-11-28

**Authors:** Guocheng Gao, Jingxuan Liu, Mingyue Xu, Rui Xia, Lunan Zhao

**Affiliations:** ^1^School of Sports Science, Qufu Normal University, Qufu, China; ^2^Physical Education College, Shandong University of Finance and Economics, Jinan, China

**Keywords:** China, youth, promotions of physical activity, policy agenda, policy transformation

## Abstract

Promotions of physical exercise for adolescents have become key elements of the country’s national sports and health campaigns in China. Specifically, these promotions have gone through four stages including Initial Institutionalization, Standardization and Legalization, Solidification and Publicization, and Comprehensiveness and Diversification, which are interpreted based on the Multiple Streams Framework with discussions of the role and dynamism of the problem streams, political streams, and policy streams included. The results reveal that the political streams, reified by the will of the governing party and the central government in particular, play a leading role in policy transformations. Consequently, identifications of existing problems and subsequent adoption of proper measures emerge as the key to generating policy developments in the promotion of physical exercise for adolescents in China.

## 1 Introduction

Physical inactivity is the fourth leading risk factor for mortality globally and is responsible for 6% of deaths worldwide and for around 10% in the World Health Organization (WHO) European Region (1). According to the Report on the Development of Youth Sports in China published by the State General Administration of Sports and a large number of studies, the age range of the youth group is roughly 6–19 years old (2). The habit of physical activity in childhood and adolescence reduces the risk of diseases such as cardiovascular disease, overweight and obesity, diabetes and cancer, and also positively affects a person’s physical and mental health, and even intellectual and social wellbeing (3–5). In China, promotions of physical activity for adolescents (PPAA) have become key elements of the country’s national sports and health campaigns, as manifested by the inclusion of children and adolescents’ participation in sports in the recent “Outline for Building a Leading Sports Nation” and the “Healthy China Initiative 2030,” foregrounded as part of the important national development goals (6, 7). However, the latest report on the physical fitness of adolescents in China indicated a severe gap between policy and reality. On physical attributes alone, the obesity rate of male adolescents (aged 7–22) has skyrocketed, increased around 24 and 44 times for those living in urban and rural areas, respectively, in the last three decades, and a relatively mild yet still alarming figure was also witnessed for their female counterparts, which saw a 12-fold increase in obesity rate (8). Meanwhile, other physical attributes of the adolescents in China, such as fitness and eyesight, kept declining (9). Another report revealed main contributing factors that impede adolescents’ participation in physical activity, including replacement or cancelation of physical education, imbalance between supply and demand for extracurricular sport services, lacking of organized and professional guidance for adolescents in sports, and the more extreme mentality of “fearing sports will damage one’s academic performance,” all of which have highlighted the more pronounced negativity towards sports and physical activity for the adolescents in China (10).

In actuality, the emphasis on PPAA in physical activity can date back to the founding of the People’s Republic of China (PRC) (7), discussions on this topic have always taken place in the highest leadership of the country, from the Political Bureau of the Central Committee of the Communist Party of China (CPC) to the Executive Meeting of the State Council, and have materialized as legislations, regulations, and implementations of Party policies. Relevant policies have undergone several “milestone” reforms and updates, such as the modification of the traditional “top-down” model of physical education for adolescents and its eventual ascension to national level policies (11). However, the result never fully met its expectations, regardless of the policy support. For a long time, relevant work on PPAA and the current status-quo of the physical fitness of the adolescents have been tangled by multiple factors including the country’s process of economic development, political landscape, education, and social context (12). Other more salient issues including the lack of actual content and sound structure of the policies, their compatibility with the actual situation, have also posed negative impacts on the proper implementation of the promotion (13). The broader economic, political, cultural, and social elements, mixed with the more salient policy-wise issues, have led to a reduced “chemistry” between reality and policy in the country’s efforts to promote physical activity for its adolescents. Thus, this situation propelled us to ask the following questions:

*RQ1*: What factors attributed to the constant increase of attention from the government and the CPC on policies relevant to PPAA?

*RQ2*: What are the “key events” that have led to the reforms and innovations of the policies?

*RQ3*: Why does the current status-quo of the adolescents’ physical fitness fail to meet the expected result in the policies?

These questions will be answered by reviewing the procedural elements of the policies on PPAA in China and the key events centered around these policies. The evolvement and dynamic mechanism of the policy reforms and updates with the purpose of providing references to advocates of PPAA will be analyzed through historic reviews and Multiple Streams Framework, solutions to physical fitness problems of the adolescents from sports, and other major public health policies, thus more effective actions could ensue when possible “Policy window” surfaced.

## 2 Multi source flow theory analytical framework

How public issues attracted the attention of policymakers and are gradually included in government agendas has always been one of the focuses of studies on policy sciences. Centering on “why does the policy change,” multiple policy analysis theories were introduced since the establishment of this discipline. Earlier scholarly literature on this front were mostly vested in the Rational Choice Theory, including Decision Theory, New Institutional Sociology, Social Choice Theory (14). However, questions on the efficacy of the Rational Choice Theory and the subsequent rise of the Concept of Bounded Rationality have led to a new breed of theories including Advocacy Coalition Framework Theory, Punctuated-Equilibrium Theory, Policy Diffusion Theory (15), mostly notably the Multiple Streams Framework (MSF) from Kingdon and Stano (16), which was built on Cohen’s “Garbage Can” mode (17). The MSF stands as a new analytical framework of policy process based on complexity theory and new institutionalism aimed at the establishment of agendas and public policies, which has become one of the most important theories in public policy studies (18). The MSF has focused on the problematic preferences, political agenda setting, and policy choices of policy analysis, which are reified as problem streams, political streams, and policy streams respectively, with each of these factors following its own dynamic characteristics and rules (19).

First of all, the problem stream is composed of various problems in the society, which refers to the focused attention of the government and policy makers due to specific problems, which are highlighted through a series of quantitative indicators, immediate feedback of the real situation and the focus events that trigger widespread concern in the society, prompting the establishment of the policy agenda, which is the starting impetus in the process of policy making, and how the problems that exist in real-life scenarios and have a wide range of public interest It is the process by which issues of broad public interest that exist in real-life scenarios are brought to the attention of the government. Other factors in the problem streams include major identifying indicators, negative policy feedbacks, or capabilities of policy authorities on handling issues, etc. (20). To wit, the analysis of problem streams aim to identify public social problems and investigate the reasons behind why certain issues gathered the interest from government officials and propelled them to include these issues into government agendas.

Secondly, policy streams are diversified policy ideas and solutions from different policy communities around a certain social issue, and based on their respective professional perspectives and interests, they compete to put forward innovative policy ideas, providing a rich pool of alternatives for policy formulation.

Finally, political streams refer to political processes that affect settlement of issues. Such processes include the Party’s Ruling Philosophy, the shift of political powers, emotions of national citizens, pressures from interest groups, and public opinions. Among other things, the Party’s Ruling Philosophy and the shift of political powers often greatly influences the formulations of policies.

The said three streams would gradually develop and confluence at specific event or time juncture, opening a “policy window” for public issues to be included into policy agendas and subsequently drive policy developments and policy updates (21). The confluence of the three streams exemplifies the dynamic integration of specific issues, policy proposals, and political landscapes. This integration, plus the exterior “push” from “policy entrepreneurs” and the opening of “policy windows,” jointly contributed to the establishment of policy agendas. In short, the MSF demonstrates a clear theoretical structure and strong explanatory power for it believes that the mixture of problem identification, feasibility plan, and political environment is the crucial factor for the formulation of policies, and thus unlocked the “black box” of policy-making. The MSF reveals multiple elements that drive the introduction of certain public policies at a specific time juncture and serves as a scientific mode of proof of the rationality in policy-making and drivers of policy changes. It is characterized by understanding the intricate and interactive logical mechanism between various influencing factors in the process of policy agenda setting, which provides a thinking path and theoretical support for in-depth analysis of the formation and evolution of public policies. Admittedly, the differences between political systems also make prominent limitations of adopting the MSF indiscriminately (22, 23).

Since the multiple streams theory was born in the West, China has a different political system than the West, with the former being more centralized, which often leads to differences in political streams. Therefore, in the Chinese social environment, the ideology and ruling philosophy of the CPC plays a key leading role. Specifically, the absence of interest groups and party turnover in China’s political system, the special position of the CPC as the leading center of the socialist development path with Chinese characteristics and as the only ruling party in China, and the role of government agencies as the main body of policy formulation and implementation, make it possible for China’s political streams to remain dominated by the Party and the government. As a result, the ruling philosophy of the CCP has become a key element of the political stream, and its policies are often implemented in line with the ruling party’s ideology, with the political stream mostly epitomizing the will of the top leadership (24). When confronted with the issue of youth sport and health in China, successive generations of party and state leaders have had a consistent line of thought, all of whom have attached great importance to the top-down supply of youth sport and health policies, which has profoundly influenced the construction and improvement of youth sport and health-related policies at various stages of historical development. At the beginning of the founding of the People’s Republic of China, the Communist Party of China (CPC), while comprehensively refreshing its political construction, economic construction, social construction and Party construction, got rid of the old and brought in the new and vigorously strengthened its ideological construction. Comrade Mao Zedong’s educational tenet of “health first, study second” became a slogan and catchphrase widely promoted throughout the country. Youth sports and health policies have arisen and developed in this context, and have also profoundly influenced the construction of the subsequent relevant policy system. However, the backward concepts of modern society and the backwardness of the country’s economic conditions may have led to a one-sided understanding of the goals of education; and the excessive pursuit of success, with an emphasis on political campaigns, productive labor, and the development of the body, may have neglected the systematic and professional nature of physical education, resulting in youth The diversity and scientific nature of physical exercise is insufficient. Subsequently, Comrade Deng Xiaoping put forward the idea that “we should strive to make our young people idealistic, moral, knowledgeable and physically strong” (25), and Comrade Hu Jintao put forward the “people-oriented” scientific concept of development, and made the important decision to strengthen the sports and physical fitness of young people; these policies have contributed to the continuous development and improvement of China’s youth sports and health policies. However, at the same time, the excessively fast pace of economic and social development has brought about a series of negative problems, including a lack of attention to physical activity and physical health among young people, as well as a series of problems such as declining vision and obesity among young people, which have accompanied modernization. Currently, China is in the Xi Jinping era, and as the problem of youth physical health has seriously affected national and social development, youth sports and health promotion policies have been given unprecedented height and depth, stimulating widespread concern and participation in youth sports and health by the whole society. However, the specific policy measures and the implementation of the policies remain to be examined. In the process of policy formulation and implementation, the leadership of the Communist Party of China (CPC) and the leading role of governmental agencies are obvious; the youth sports health promotion policy has been continuously separated from the old policy and deeply integrated with the new era, and the introduction of the policy at each stage has profoundly embodied the ruling philosophy of the leaders, which has had a multifaceted and multi-layered impact on the youth sports health policy.

**Figure 1:**
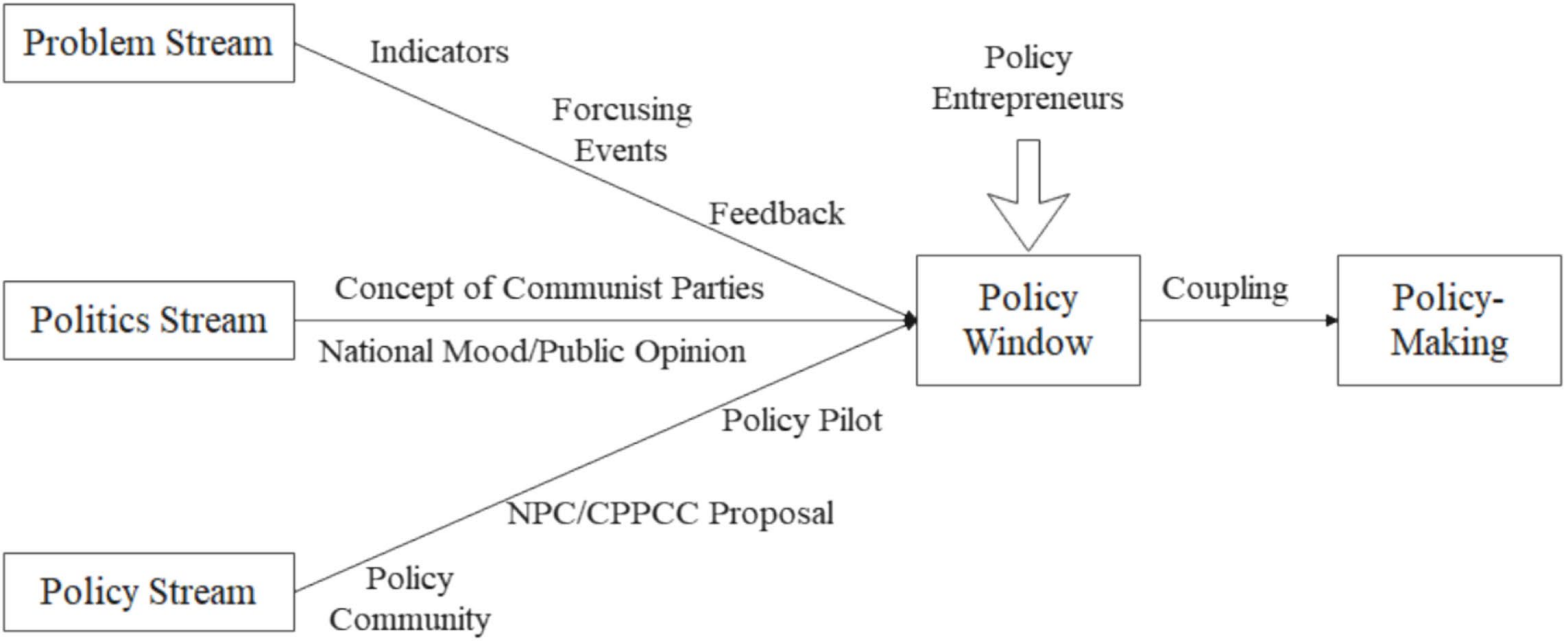
A modified model of MSF.

Another difference lies in policy streams and coupling of streams. The National People’s Congress (NPC) and Chinese People’s Political Consultative Conference (CPPCC) are the major policy communities that participate in the policy agenda settings under China’s political system (26, 27). After the problem streams invoked shifts in national opinions and subsequent attention from the government, the policy communities would provide suggestions and solutions, which are later converged into the coupling of streams. To wit, the policy streams were integrated into the coupling in the first window opened by the problem and political streams, which laid the ground for the opening of the second window of opportunity, allowing the issue to enter the policy agenda. Consequently, the order of appearance of the three streams in China is almost predictable, which follows a problem-political-policy sequence (24), making possible top-down policy reforms. Moreover, the policy streams under China’s national circumstances involve three additional modalities. The first is the local pilot projects that follow a bottom-up pattern, since if certain policies introduced by the central leadership received success in a local region, then they would possibly be able to be implemented elsewhere in the country given China’s centralized political system. The second modality involves suggestions and proposals from department directors and experts and scholars. However, all of these increments to the policy streams are affected by the ideology of the governing party as they need to be consistent with the fundamental political interests. Consequently, it is necessary to take into consideration the leading role of the ideology of the governing party and its extensive implications. Due to the reasons listed above, it is necessary to make subsequent modifications to the MSF to make the theory more relevant to China’s policy practice, as shown in Figure 1.

Under the modified MSF theory, this study aims to identify drivers for policy changes of PPAA in China through discussions of previous policies. More specifically, studies of the problem streams focus on the analysis of events that affect the adolescents physical fitness and feedbacks to existing policies with “Chinese National Survey on Students” Constitution and Health (CNSSCH) as indicators; analysis of the policy streams, based on the understanding of the problems, discuss major pathways of how “policy primeval soup’ is created by national leaders, government officials, scholars, and practitioners; discussions of the political streams include opinions of national citizens, ideology of the CPC and the top leadership, and public opinions from generated by the media, with a major focus on the delineation the concepts and ideologies of national leaders as they shape the political environment for policy changes.

## 3 Methods and data

The long-term span of the policy makes it difficult to conduct “interviews on events that lead to policy development, policy entrepreneurs, and processes (15).” The process of research methods includes three steps. The first step includes policy reference materials that are directly related to adolescents, physical exercise, health promotion, and population-wide policy. On CNKI, the literature searches are used to use the words “youth policy,” “physical exercise policy,” “health promotion policy,” and “population-wide policy” on CNKI. From 1949 to 2020, 341, 290, 664, and 320 references were obtained. The theme included sports and finally screened 115, 290, 62, and 65 references. A Google Scholar search using the terms “youth policy,” “physical activity policy,” “health promotion policy,” and “population-wide policy” yielded 3,830,000, 15,900, 5,980,000, and 4,670,000 results, respectively. The search using the terms “youth policy,” “physical activity policy,” “health promotion policy,” and “population-wide policy” yielded 3,830,000, 15,900, 5,980,000, and 4,670,000 results, respectively. A similar approach applies to other search engines. A phased review strategy was adopted, starting with an exhaustive review of the information embedded in the abstract section, followed by verification of the sources and channels of publication of these documents, and finally delving into a meticulous review of each of the materials. The first step produced 285 journals, 224 scholarly articles, 2 newspapers, 1 book, and 29 conference articles that served as the basis for the next step.

The second step was to search for keywords (youth, sport, physical activity, health promotion, and population-wide policy) in digital databases of official national documents, including the Sports Laws and Regulations Document Retrieval System,^1^ the China Laws and Regulations Database,^2^ and the State Council Policy Documents Database,^3^ etc. From these sources, a wide range of policy documents can be obtained, including legislative acts, policies, initiatives, action plans, opinions, decisions and circulars. The data-set also includes official statistics, transcripts of speeches by national leaders and government officials, annual reports on youth development and news reports. With the restriction of time frame and the utilization of precise query, 187, 9, 50, 3, and 1,903 references were obtained in the Sports Laws and Regulations Document Retrieval System, 1, 56, 0, 3, and 28 references were obtained from the Chinese Laws and Regulations Database, and 4, 151, 1, 3, and 10 references were obtained from the State Council Policy Documents Database. The second step produced 60 statutes, 82 documents, 87 bulletins, 9 policies, and 2,143 reports that served as the basis for the next step.

The third step of screening the literature obtained above is a gradual process of screening the literature that is most relevant to the topic of the study, based on predefined inclusion and exclusion criteria. Specific steps include an initial screening to eliminate irrelevant and duplicative literature, followed by a more in-depth assessment of the remaining literature to ensure its quality and applicability. After completing the data assessment, analysis and presentation, 384 PPAA related policy documents were finally selected to support the article, which were within our target range of keywords and time and analyzed according to the research objectives with the help of MSF.

## 4 Results

### 4.1 Document quantity analysis and policy stage division

**Figure 2:**
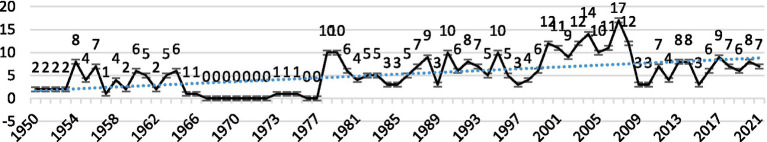
Numbers of changes of PPAA policies since the founding of New China.

Figure 2 shows the time sequence of collections of documents. From 1949 to 2021, 384 policies relevant to PPAA were introduced (5.2 annually). The quantity of the policies revealed a generally increasing trend, indicating the constant rise of importance attached to PPAA from national authorities. Yet the annual quantity of policies shows a relatively high dispersion rate (3.87 standard deviation), which means high policy fluctuations. A closer look at the trend of fluctuation indicates the quantity of documents spiked at 1954, 1978, and 1999, and plateaued between 1999 and 2008, followed by a sharp decline in 2011 and a subsequent stable growth since 2012. The dynamic change in the number of policies introduced annually is highly related to influence of the three streams. Based on the historical lineage, social background, leadership changes, and the temporal characteristics of policy dynamics, the policy history of youth sports and health promotion is roughly divided into four stages: the initial institutionalization stage (1949–1977), the standardization and legalization stage (1978–1998), the solidification and publicity stage (1999–2011), and the synthesis and diversification stage (2012-present).

The first phase (1949–1977) is characterized by Initial Institutionalization. This period is in the dawn of the new China and the 10 years of the Cultural Revolution stormy and shaky intertwined with the complexity of the period, in the political strife and chaos and other unfavorable situations, from the central government to the local sports department of the entire sports front into paralysis, the sports business basically stopped, but in the Party Central Committee and the State Council’s care, the youth sports policy quietly sprouted, embarked on the preliminary stage of exploration. During this period, its main purpose was to safeguard the physical health of young people, and the content of the policy was relatively homogenous, focusing on enhancing the physical fitness of students and promoting physical exercise, with the Government oriented towards the prevention of disease and the improvement of physical fitness through physical exercise, reflecting the guiding ideology of “health first,” and serving the goal of improving the physical fitness and health of young people. However, specific implementation measures and systems are not yet perfect, and the development of school sports is mostly promoted through administrative means. The Ministry of Education, the National Sports Commission and the Ministry of Health, among others, have jointly issued a series of policies, represented by major policies including “The Decision on Improving Health Conditions of Students at Various Levels of Educational Institutions (1951)” and “Regulations of Labour and Defence (1958).” The policies introduced in this stage focused on “disease prevention and improvement of physical fitness.” With school physical education as main field of interest, the initial physical education system in China was established, with curricular content such as classes and textbooks and extracurricular activities including sports competitions, radio broadcast exercises, and system of labor and defense. This stage marks the beginning of the model of PPAA featuring the integration of curricular and extracurricular contents centering around school physical education.

The second phase (1978–1998) can be seen as the stage of Standardization and Legalization. With the advancement of reform and opening up, youth sports policy has entered a phase of recovery and reconstruction, and the policy system has begun to focus on guaranteeing the smooth implementation of youth sports activities through institutionalized and legalized means, thereby promoting the comprehensive and healthy development of young people. With the introduction of major policies including “Interim Work Regulations of Full-Time Primary and Secondary Schools (Draft) (1978),” “Regulations on the Work Concerning Physical Education and Sport in Schools (1990),” “National Fitness Guidelines, and Law of the People’s Republic of China on Sports (1995)” during this period. The introduction of the document marks the youth sports work into the legalized track. There is an important legal guarantee for the standardization and scientific development of school sports. Although still focusing on school physical education, the policies in this era propelled the establishment of specific legal regulations, and have become more inclusive in terms of subjects, including physical education, extracurricular activities, after-school sports training, sports entrance examination, faculties of physical education, organizational structure and management, facility, equipment, device, and funding. Moreover, this stage also saw the addition of the national system of physical fitness monitoring and examination for adolescents, manifested by routine surveys and close monitoring and the “health card” database for the physical fitness and health of adolescents, and the construction of a more complete system of physical education and sports policies for adolescents.

The third phase can be seen as the stage of Solidification and Publicization. With the continuous improvement of the policy system, the youth sports policy has gradually solidified, forming a more stable policy environment, and the policy has begun to focus on the public nature of youth sports, promoting the tilting of public sports facilities and services in favor of young people, and improving the participation of young people in sports activities as well as the accessibility and fairness of sports resources. It focuses on policies such as “The Decision on Deepening Educational Reform and Comprehensive Promotion of Quality Education (1999),” “Outline of the Reform and Development of Physical Education 2001–2010 (2000),” and “Opinions of the CPC Central Committee and the General Office of the State Council on Improving Physical Fitness of Adolescents (2007).” The policies in this stage elevate PPAA to national level strategies, which saw the introduction of an interconnected physical activity promotion network (e.g., the “Home-School-Society” initiative). The focus of these policies was thus on promoting the establishment a social support network and incorporate more social groups into the promotion agenda of physical education and health for adolescents through setting up dedicated sports clubs and pilot projects for outdoor sports camps for adolescents. The introduction of the new “curriculum-extra curriculum” and “on campus-off campus” models created important institutional assurance to the fostering of a supportive social environment for the development of practice of physical activity and health for adolescents.

The fourth phase can be seen as the stage of Comprehensiveness and Diversification. Youth sports policy has entered a stage of comprehensive and multifaceted development, with more comprehensive and systematic policy content, covering a wide range of areas such as sports, education and health. The policy orientation gradually focuses on synergistic cooperation among the Government, schools, families and society to jointly promote the development of youth sports. The Notice on “Notice Concerning Further Improving Work on School Physical Education (2012),” “Opinions on Improving the Role of School Physical Education in Promoting the All-around Development of the Physical and Mental Health of Students (2016),” “Guideline for Healthy China Initiative 2030 (2016),” “Promotion Plan for Physical Activity of Adolescents (2017),” “Outline for Building a Leading Sports Nation (2019),” “Opinions on Promoting Healthy Development of Adolescents through Deepening Integration between Sports and Education (2020)” were adopted. Building upon the school physical education system and the off-campus public sports services for adolescents, the policies managed to set up a comprehensive governing structure for school physical education featuring “policy-implementation-assurance-monitoring-evaluation-feedback” and a diversified off-campus public sports service structure which is led by the government, and jointly built by the authorities and the general public, covering areas including academic educations, sports activities, health promotions, sports organizations, and facilities. Youth sports policies and health promotion policies were highly integrated during that period, working together to build a comprehensive system of youth health promotion.

### 4.2 Policy transformations of PPAA in China from the perspective of the MSF

#### 4.2.1 Problem streams

Problem streams lead to the establishment of new policy agendas and propelled shifts in policy landscapes. During the development of PPAA in China, the problem streams witnessed in the various stages of this process drove the adoption of new proposals, which subsequently resulted in policy overhauls once supported by top leadership.

The first stage of the said process can be located at the beginning of the founding of the PRC. Tormented by long-term warfare, economic depression, unfavorable medical conditions and the Cultural Revolution. The political movements and struggles of this period disrupted the normal educational order for young people, who were unable to receive systematic education and training (28). Although the Government has enacted a number of youth policies to protect and promote the healthy growth of young people, the effectiveness of the implementation of these policies has been affected to a certain extent due to the special background of the Cultural Revolution, resulting in the relatively poor physical fitness and health of young people during that period. Issues including maldevelopment, physical impairment, and high morbidity have seriously affected the enrolment and employment of adolescents, with around 8.1% students suspended schooling due to illness (29). The prevailing social climate has, to a certain extent, influenced the values and behaviors of young people, which may lead to the establishment of biased worldviews and values, adversely affecting their moral character and sense of social responsibility, and in turn affecting their physical fitness behaviors (30). The scarcity of appropriate policy structures, together with a general lack of concern to improve the fitness and health conditions of adolescents for the ever-increasing social, economic and labor requirement, have surfaced as main reasons for China’s efforts in its Initial Institutionalization of policies of PPAA.

The second stage of the development of promotion policies took place after the Cultural Revolution, with impacts from major crises being one of the main factors that drive policy changes. Under this historical context (31), policy structures established during the first stage have gone through drastic changes, including the replacement of PE curriculum with labor and military training, and even the cancelation of PE classes (32, 33), all of which have greatly damaged PPAA. Reports showed that 53% elementary schools, 73% junior high schools, and 94% senior high schools had either limited hours of PE classes or even canceled them entirely, and more than 30% of schools failed to maintain regular physical activities for students (34). Thus the promotion policies at this stage mostly focused on recovery and reconstruction, as well as institutionalization and law-based practices.

Policy shifts in the third stage can be mainly attributed to changes in key indicators and subsequent policy feedback. In 1985, China launched its first CNSSCH on a national scale. Fast-forward to 2005, the results revealed a 20-year consecutive decline in the fitness and health conditions of adolescents in China (35), exposing the inadequacy of governance in this respect. Researches at this time period revealed a general weak health awareness, low interest in physical activities, and negative attitudes toward sports among Chinese adolescents, often reified as long screen time (36, 37) and mounting pressures in an exam-oriented education (38, 39), which were caused by a mixture of elements including rapid economic development, lifestyle changes due to social and technological transformations (40), misplacement of physical education orientations, and mismatch between curriculum and reality (41). As the government has been preoccupied with economic development, fewer attentions were allocated to public issues, which led to the increasing prominence of previously neglected social hotspot issues, such as the lack of sports facilities, faculties, and services (42). Consequently, the point of focus of this stage dwells on the rectification of the negative effects from previous policies and relevant modifications, which led to the readjustment of the scope and focus of governmental functions.

The latest stage of the policy development coincides with the shift of China’s economy from the previous “high speed” growth model to the current “high quality” growth model, and policy feedback have become the main driver for policy transformations. The shift of growth models and the top-level design (43) means the abandonment of the previous “extensive” development model of the promotion policies. However, relevant governmental functions failed to undergo fundamental transformations. Specifically, the absence, misplacement, and overreaching of certain governmental functions remain unresolved, and the “administrative barrier” between government departments remain unbroken, which greatly stifled the collaborative advantages between departments (44). The collisions between the old and the new policy structures during reforms and transitions limited market vitality and public creativity, overshadowing the benefits and functions of school physical education and public sports service. These issues have propelled the promotion policies during this stage to re-shift their focuses on the modification of ecology of school physical education and the enhancement of public sports services.

#### 4.2.2 Political streams

**Figure 3:**
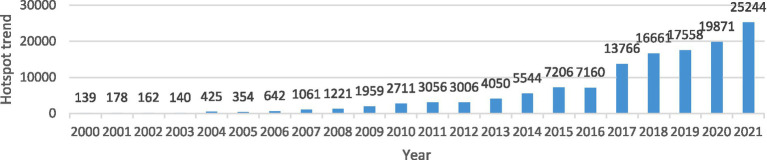
Hot-spot trend of news on the health of adolescents in China News Database.

Political streams often play decisive roles in directing the transformations of policies of PPAA in China. Factors including changes in public opinions and new governance concepts usually cause large-scale policy transformations. Changes in public opinion do not only reflect the general appeals and attitudes of national citizens in policy agendas, but also affect the establishment and implementation of policies. Based on the statistics from China News Database,^4^ joint focus, reflection, and identification on health-related topics for adolescents emerged as key elements in public opinions that push policy changes, and discussions about the physical health of young people are increasingly at the forefront of public debate, driven by the media (Figure 3) (45). However, under the current socio-cultural context in China, policy structures of PPAA also ignited fierce social debates. Due to the unbalanced focus on entrance examinations (e.g., college entrance examination, or Gaokao) and academic curriculum, most parents believe that physical activity would damage their children’s competitive edges in their academic performance (46–48). This public opinion of weighing academic courses over physical activity in an exam-oriented education system (49) greatly impeded the establishment and implementations of relevant promotion policies. Consequently, in order to remedy the status-quo of favoring academic achievements over physical wellbeing, recent policies of PPAA have foregrounded the inclusion of school entrance examinations as the new focus of policy orientations (50). The authorities expected to raise public awareness of promotions of physical activity through the introduction of sports exams and this policy inclination continues to intensify.

Under China’s political system, governing strategies of the CPC appear as the political backdrop that delimits that orientation, objective, and contents of the policies of PPAA. Meanwhile, the establishment, implementation, and transformations of such promotion policies also reflected the shifts of CPC’s governing strategies, becoming one of the key driving forces of policy changes.

The first stage of the policy development took place when the main focus of the CPC was economic recovery and the consolidation of administrative power and national defense, with China adopting the model of planned economy from the Soviet Union (51). The concept of Mao Zedong’s New Democracy has incorporated sports activities as one of the indicators of the political success of the Socialist revolution and the Communist Party (52). During this time period, the will of the leadership that affects the policies of PPAA can be summarized as “serving labor productivity and national defense,” “developing sports activities to enhance fitness of national citizens,” and “learning from advanced experience from the Soviet Union” (31, 53). This has directly resulted in highly centralized, military-oriented, and Soviet-based policy structure that was highly unrealistic, rigid, unified, and planned, which can be epitomized from political tendencies of “replacing sports with labor” and “replacing sports with military training” (54), as well as the “copy and paste” of Soviet style curriculum and teaching materials of school physical education. This has become the foundation of the policies of PPAA centered around school physical education in China during this stage.

The Second Plenary Session of the 11th Central Committee of the Chinese Communist Party marked the beginning of the second stage of the policy development as China entered its era of Reform and Opening up and modernization, during when the CPC Central Committee proposed a renewed education guideline featuring “Prioritizing Educational Development” and “Fostering All-around Development for the Students,” which redirected the government’s focus back to school physical education. Dedicated organizations and working structures were established by educational departments with the introduction of a series of special laws and regulations on school physical education and evaluation mechanisms for sports-related curriculum, faculty, and facility were also in place. The resolution of “Deepening the Reform Comprehensively” from the Central Committee of the CPC further diluted the “highly centralized” management model of physical education in China, and marked the beginning of the shift of governmental functions in this area featuring a streamlined and layered management structure (31). Under the initiative of establishing a national fitness program centered around adolescents (55, 56), this revamped system of physical education in China signified the start of a market-oriented and rational approach toward the sports industry in the country (57) and paved the way for future expansions of the policies of PPAA in the next stage.

The policies introduced in the third stage happened during when the central leadership proposed to “establish and improve the socialist market economy,” a relatively key moment in China’s development. Thus, the main objectives of the policies of PPAA were redirected to conform to the new economic outlook in the country. With the establishment of national development plans including, “Quality-oriented Education,” “Scientific Outlook on Development” and “Strategy to Make China a Talent-Strong Country,” the government expanded its understanding of the functions of sports to areas including education, cultural exchange, politics, economy, and social activities, marking the return of “human-centered” physical education in China and a general shift of policy concepts toward the need of adolescents. Consequently, policies in this stage revealed an inclination of foregrounding balanced and rational development of PPAA, with more attentions paid to their needs in practicing sports. Moreover, the policies also highlighted a hybrid development model combining both the “Health First” school physical education system and the market-oriented public sports service system for adolescents (58). Meanwhile, the 16th National Congress of the CPC proposed a shift of governmental functions from microscopic management to macroscopic readjustments and controls, which include economic regulations, market supervision, social management, and public service (59). In 2007, former President Hu Jintao called for the meeting of the Member of the Standing Committee of the Political Bureau on discussing the fitness of adolescents (6), which led to a renewed focus of the promotion policies on creating favorable social environments (60) beneficial to facilitating sports-related health behaviors for adolescents. Through incorporating multiple interest groups (clubs, organizations, training camps, communities, families, etc.) into the policy agenda, the boundaries set for the promotion policies were further expanded from schools to the society.

The latest stage of the policies took place when President Xi Jinping announced that China has entered the “new era of socialist development.” Under this context, a series of sports-related policies were introduced, represented by “Building a Leading Sports Nation” (61, 62), “Healthy China 2030 Initiative” (63), “Nationwide Fitness Program” (6), which pushed physical activity of adolescents to a new height in the country’s general national development strategy, and a new wave of policies were implemented. Since the 18th National Congress of the CPC, the Central Committee of the CPC continued the approach of streamlining administrative powers and enhancing the efficiency of the government, exemplified by the decision on “the modernization of China’s system and capacity for governance” proposed during the Third Plenary Session of the 18th CPC Central Committee (64). The 19th National Congress of the CPC saw the introductions of principles including “building a service-oriented government that the people are satisfied with” and “fostering a social governance pattern featuring joint contribution, participation, shared benefits.” Under this context, the policies of PPAA also saw their transformations with the deepening of the reform of “streamline administration and delegate power, improve regulation, and upgrade services,” and the governance model of physical education for adolescents characterized by “government-society-market collaboration and family-school-community linkage” was gradually adopted (65), emphasizing on providing an all-around, diversified, and high-quality sports service structure to adolescents (66).

#### 4.2.3 Policy stream

When a certain social issue caught the attention of a large number of policy participants, the policy community, which consists mostly of academicians, third-party organizations, and government officials, would debate and discuss the issue through official or unofficial channels, producing “policy primeval soup” including research results, policy consultation reports and solutions for the reference of policy-makers, which ultimately shapes the formulation of authoritative and legal policies. At the initial stages of the first and second phases of policy formulations, due to the immaturity of the initial batch of policies and potential influence from underlying crises, the “policy primeval soup” mostly originates from suggestions from governmental actors who participated in the making of the policy and results from pilot projects led by local-level government departments. For example, in 1953, suggestions like “imitating the Soviet model” and “quickly establishing and improving sports commissions at various levels” from He Long, the then director of the Sports Commission of the CPC Central Committee, were approved and implemented in the 205th council meeting of the State Council in 1954 (67). Apart from work reports from relevant high-level officials, the National Sports Commission would submit policy suggestions proposed during its annual meeting to the central government in forms of meeting minutes or reports, which were later adopted as policies and implemented after modifications from the decision makers in the central government. However, the adaptability of certain innovative policy proposals or suggestions in actual practices remained unknown at the beginning, thus requiring the government to allocate available resources to conduct empirical studies of the effects of the policies, or even pilot projects. These local level policy experiments and innovations often served as indicators for the national government to regulate and readjust the policies adopted. For instance, the “Ready for Labor and Defence” was initially tested as pilot projects in the primary and middle schools in Beijing, Shanghai, and Tianjin and other major cities in 1951, was later modified and implemented as the “Regulations of Labor and Defence” in 1958 and re-adapted as the “Physical Training Standards for Adolescents” in 1964 (68).

In the third and fourth stages of the policies, as the fitness and health issues of adolescents intensified, policies of PPAA were elevated to the status of national strategies. The surge of policy participants led to more diversified sources for policies. Research reports, among other academic contributions from experts and scholars, began to provide important policy support to the opening of the “policy window,” as can be seen from the plethora of research projects aimed at promoting physical activity of adolescents funded by The National Social Science Fund of China since 2007 (69). The accumulations of research gave birth to a number of innovative and forward-looking policy alternatives and policies from the NPC and CPPCC mechanisms also greatly enriched the “policy primeval soup.” Moreover, a portion of policy entrepreneurs possesses a “dual identity” as they are both elite-level representatives in the sports industry and the political realm, thus giving them the power to provide policy suggestions for national deliberations during the opening of the “Two Sessions.” Once their proposals on the policies of PPAA were approved, these proposals would often instantly acquire legal bindings. Plenty of examples of this nature can be witnessed in recent years, such as “increasing the weight of sports in school entrance exams at various levels” proposed by Dai Liyi, vice president of the Huadong Normal University, Ding Shizhong, Chairman of the board of directors of the Anta Group, along with several other contributors (70). Yao Ming, the former Chinese basketball star and the current president of CBA, also participated in the policy-making process in this area and helped facilitate the implementation of more effective policies at higher levels (71). These policy suggestions also shaped public opinions on sports and made possible the suggestions from the policy community to popularize and eventually become a public consensus.

## 5 Discussion

### 5.1 Opening of the policy window: policy transformations from the convergence of the three streams

In this paper, we explore the evolution of youth sport and health policy in China through the modified MSF theory. The declining physical fitness and the frequent occurrence of key events in China’s youth health constitute the original driving force for the emergence of policy issues; the reality of the problem attracts the attention of policy makers from all walks of life, and the bills, research reports, and policy recommendations put forward by the policy community around youth sports and health promotion converge to form a policy source stream, providing sufficient reference for policy changes. Most importantly, in this process, the party and state leaders’ great attention to and clear instructions on youth sport health promotion have influenced the direction of youth sport health promotion policy provision from the level of national leaders, injecting a political stream of policy change. When the three streams are intertwined and coupled, the key “policy window” will inevitably come into being, forming a powerful synergy to promote policy change.

**Figure 4:**
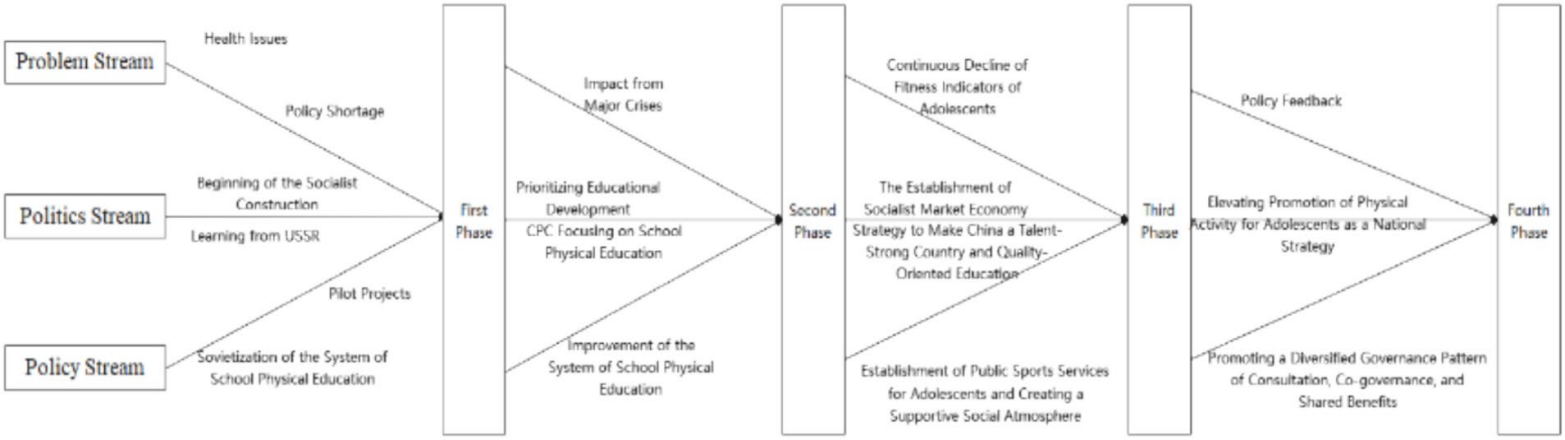
Transformations of the policies of PPAA in China based on MSF.

The results of this study are generally consistent with the findings of most studies that have explored in depth how the institutional environment shapes the paths and outcomes of policymaking through specific cases, demonstrating the unique strengths of the multi-source flow theory in revealing the complex dynamics of the process of parsing policy change, predicting policy trends, and guiding changes in policymaking. For example, changes in adolescent health-related promotion policies in Iran are mainly influenced by policy streams (72), with the issue stream in the UK taking a central role in the development of relevant policies (73–75), policy changes in the US are the result of the combined influence of the issue stream, the policy stream, and the political stream (76). China is influenced by the actual national conditions and political system, and the political stream plays a key leading role in its PPAA change process. Although, the reform and development of the multi-source flow theory policy is the result of the joint efforts from the governing party, government, society, and general public, and their ideological approach to youth sport and health promotion policy is the core of driving the policy change. The MSF believes that the opening of the “policy window” offers opportunities for policy transformations, the key to which lies in the confluence of the problem streams, the policy streams, and the political streams (77). The dynamic interactions of the three streams induced noticeable changes and modifications of the objectives, focus, concepts, modes, scopes, and requirements of such promotion policies in China. If interpreted from the perspective of the MSF, salient issues concerning the fitness and health of adolescents can be seen as rationales for policy transformations, governing concepts and wills of the leadership at different time periods serve as policy orientations, and proposals and suggestions from members of the policy coalition provide the legal foundation for the changes in the policy. In summary, the “policy window” opened during the interactions and integration of the three streams, which led to the transformations of the policies of PPAA in the country (Figure 4).

The decision-making agenda is the key to materialize results from the “policy window” as actual implementations of policies and often dictates the effectiveness of such policies. The problem and political streams serve as crucial drivers during policy transformations. Specifically, the major incentive of the development of the problem streams mainly includes the existing issues in the fitness and health of adolescents and a pressing need to increase the effectiveness of the policies. However, external pressures are not the most prominent forces that propelled policy changes in China. In other words, the policy agendas do not force decision makers in adopting new agendas through public pressures or expressions of public opinions, since the fitness and health issues of the adolescents can be traced back to the founding of the PRC. Instead, it is the identification of the problems from the governing party and the central government that pushed forward the transformation of policies, as the accumulations of the problem streams made the top leadership aware of the necessity to renew policy agendas. Thus, the political streams were the leading factor that drove policy changes. This serves as the prerequisite for the involvement (actively or passively) of policy subjects, which significantly affected the establishment of policies and strategies and has become the main source of policy streams. Under this context, PPAA in China has successfully undergone the legal process of “policy window-decision making agenda-policy implementation.” In this sense, the three streams in this process are not mutually independent as the MSF suggested, but rather jelled into a “community” under China’s unique political system. The dynamic interactions of the three streams jointly enabled the opening of the “policy window.” As the core of the political streams, the governing party of China assumed the most salient role in integrating the three streams and the subsequent appearance of the “policy window.” Consequently, the key to policy development in China lies in if the governing party could identify the issue and adopt proper measures. It is thus necessary to engage the governing party to actively identify and analyze fitness and health issues of adolescents and closely follow the effects of existing policies and feedbacks including impediments to policy implementations and unexpected results. Meanwhile, public opinions should not be neglected and proper handling between the mismatch between emphasis on academic performance in an exam-oriented education and disregard of PPAA is crucial for the policies to achieve continued benefits. The implementation of the policies could receive great support from national citizens when the general public realize the importance of policy reforms and understand the intention that lies within, and it is believed that this could become potential spots for future researches.

Aside from this, under China’s political system, policy entrepreneurs often possess multi-facet identities. They could either be scholars, entrepreneurs, university presidents, or even star athletes (such as Yao Ming), and representatives of the NPC or CPPCC at the same time, who hold the power of policy proposals. They are keen at spotting issues and could participate in the development process of the policy before the political and policy streams took shape. This diversified identity affected the mutual-independence of the three streams and played an important role in converging the said streams and softening the decision-making level, which should be considered in future studies that analyzing China’s policy agendas under the MSF.

With regard to the youth sports health promotion policy, Korea has reoriented its youth sports policy to take into account the mental, physical and social health of youth, the development of professionalism of physical education educators, and the planning and implementation of the policy (78). Japan’s youth physical fitness promotion policy has well-established implementation rules and monitoring and evaluation mechanisms, and laws related to diet and health for youth have been enacted through the collaboration of various ministries (79). Russia has developed a State policy based on the basic principles, principles and operational rules of sport for the physical, mental and spiritual wellbeing of the population, while at the same time gaining a deep understanding of the importance of sport for health at all stages of development, taking into account the views of all sectors of society, and enhancing the relevance and democratic character of the policy (80). Based on the policy experiences of these countries, Chinese policy recommendations are proposed. (1) Problem-oriented, focusing on real problems to be solved. Focusing on the current health challenges faced by the youth population, such as the frequent occurrence of mental health problems, the lack of sufficient physical activities and other real problems, ensure that policy formulation is closely aligned with the urgent needs of youth development, strengthen the status of physical education in school education, increase the proportion of physical education courses and help promote the integration of sports and education. (2) Take a political direction to optimize adolescent health issues. Grasp a firm political direction and ensure that adolescent health policies resonate with national strategies by optimizing the policy environment and strengthening cross-sectoral collaboration, with a view to promoting the all-round physical and mental development of adolescents; through Xinhua News Agency, People’s Daily and other media, national sentiment and social opinion are guided, correct policy perceptions are established, and a favorable social atmosphere is created in support of youth sports and health policies. (3) Improve the quality of policy recommendations and develop a healthy interaction between adolescent health policies. To build an open and inclusive policy formulation mechanism, encouraging the participation of experts, scholars, youth representatives and a wide range of social sectors. Through scientific research and empirical studies, it provides a solid basis for policy formulation, forming a virtuous interactive cycle of policy formulation, implementation and evaluation to ensure that adolescent health policies can be implemented accurately and effectively, and escort the healthy growth of adolescents. In addition, healthy dietary choices, extensive sports participation, and health education about physical activity in the school environment are also important factors influencing the physical fitness of adolescents, such as the United States Partnership for Healthy Schools program, which incorporates aspects of nutrition, physical education, and education (81). Therefore, it is necessary to consider comprehensively promoting a healthy diet culture in the campus environment, deepening the integration of physical education with education, nutrition and other multidimensional aspects, enhancing the community’s understanding of the importance of health education for young people’s physical activity and making timely and appropriate policy adjustments, thereby promoting young people’s health.

In conclusion, this study not only examines the applicability of the revised MSF on the transformations of PPAA in China by including country-specific political characteristics (in this case China and the CPC), but also reviews key elements and events that affected policy changes. It is thus hoped that the result could serve as a future reference for us to anticipate the opening of the “policy window” and seize brief opportunities to present policy agendas to the decision makers. Certainly, this study has some limitations. For example, most of the second-hand data of the research come from open data on official websites, and the efficacy and reliability of which might be contested as the statistical data released by the Chinese government are sometimes considered inaccurate (23). Thus, the dataset could be enriched by incorporating interviews from experts and scholars from relevant fields. However, the extended time-span included in this study (from the founding of the PRC to present day China) may have rendered this approach impossible. Nevertheless, the following questions remained to be answered. What are the key elements attributed to the underdevelopment status of China’s efforts for PPAA? How could policy agendas be expedited through modifying identified elements in policy transformations? These questions may benefit future research in this domain.

## Footnotes

^1^www.sport.org.cn/search/system/

^2^flk.npc.gov.cn

^3^www.gov.cn/zhengce/zhengcewenjianku/index.htm

^4^www.ringdata.com/news
